# Testing an educational intervention to improve health care providers’ preparedness to care for victims of elder abuse: a mixed method pilot study

**DOI:** 10.1186/s12909-022-03653-8

**Published:** 2022-08-03

**Authors:** Johanna Simmons, Atbin Motamedi, Mikael Ludvigsson, Katarina Swahnberg

**Affiliations:** 1 Department of Acute Internal Medicine and Geriatrics in Linköping, and Department of Health, Medicine and Caring Sciences, Linköping University, Linköping, Sweden; 2 Department of Emergency Medicine in Norrköping, and Department of Health, Medicine and Caring Sciences, Linköping University, Linköping, Sweden; 3 Department of Psychiatry in Linköping, and Department of Biomedical and Clinical Sciences, Linköping University, Linköping, Sweden; 4 Department of Health and Caring Sciences, Faculty of Health and Life Sciences, Linnaeus University, Kalmar, Sweden

**Keywords:** Forum theatre, Forum play, Intimate partner violence, Medical education

## Abstract

**Background:**

Elder abuse is prevalent and associated with ill-health. However, health care providers often lack education about elder abuse and older patients’ victimization often remains unknown to them. In this pilot study we performed initial testing of an educational model aiming at improving health care providers’ preparedness to care for older adults subjected to abuse, or more specifically their self-reported propensity to ask older patients questions about abuse and perceived ability to manage the response.

**Methods:**

The educational model consisted of a full training day about elder abuse, including theory, group discussions and forum theatre. Forum theatre is an interactive form of drama in which participants are not only observers, but rather spect-actors, urged to participate in the scene. They are thereby given the opportunity to discuss and practise difficult health care encounters. Medical interns (intervention group n = 16, control group n = 14) in Sweden participated in the study and a mixed method convergent parallel design was used. Quantitative data was collected at baseline and 6 months post-intervention using a questionnaire (the REAGERA-P). Qualitative interviews were conducted with four of the participants in the intervention group and data was analysed using qualitative content analysis.

**Results:**

The reported frequency of asking older patients questions about abuse increased in the intervention group (p = 0.047), but not the control group (p = 0.38) post-intervention. Potential mediators for the improvement were an increased awareness of elder abuse and higher self-efficacy for asking questions about elder abuse. Participants also reported a higher perceived ability to manage cases of elder abuse, even though uncertainties concerning how to provide the best possible care remained. The qualitative interviews indicated that learning from each other in group discussions and forum theatre likely was an important contributor to the positive results.

**Conclusion:**

This pilot test indicated that the educational model may be effective in improving health care providers’ preparedness to care for older adults subjected to abuse. However, uncertainties about how to handle elder abuse cases remained post-intervention. In a future full-scale test of the model more focus needs to be put on how to manage cases of elder abuse.

**Supplementary Information:**

The online version contains supplementary material available at 10.1186/s12909-022-03653-8.

## Introduction

The prevalence of elder abuse in community settings has been reported at 16% worldwide [1]. Elder abuse includes physical, emotional, sexual and financial abuse, as well as neglect, and it occurs at the hands of both professionals and family members, e.g. adult children and intimate partners. Elder abuse has been associated with psychological ill-health, disability, increased hospitalization and emergency department use, as well as admission to assisted living facilities [2–5]. Despite the negative health consequences, victims are often hesitant to ask for help and the majority of elder abuse cases go unreported [6, 7]. It has therefore repeatedly been pointed out that the health care system plays an important role in detecting and reporting cases of elder abuse [7, 8]. However, health care providers are often unaware that their patients are suffering from elder abuse and are often unsure how to manage cases. We previously reported that only half of personnel at an acute internal medicine and geriatrics clinic in Sweden had ever talked about abuse with an older patient and half of respondents were rather or very concerned about not being able to give victims a proper follow-up [9, 10]. A similar lack of awareness and knowledge about elder abuse has been found among health care providers in other studies, both in Sweden and internationally [11–13].

One factor found to be associated with improved recognition and management of elder abuse is having received education about elder abuse, which is also often sought after by care providers [8, 9, 14, 15]. Education needs to address known barriers and facilitators for asking about elder abuse and managing the response. Barriers have been reported on a personal level, as well as on an organizational and system level [16]. On a personal level, health care providers are often unsure of what constitutes abuse and when it should be reported [14, 17–19]. Many providers report feeling uneasy when addressing the issue, as well as lacking confidence in their ability to manage cases. Also, fear of retaliation from the perpetrator and concern about negative reactions from the patients are common among care providers, and some express concerns about difficulties in ensuring privacy when asking about abuse [14, 16, 19–21]. Time constraints and unclarity about who has the responsibility to care for victimized patients are often reported as barriers on an organizational level [14, 16]. Also, care providers do not always feel confident that the support systems can sufficiently meet the need of older adults subjected to abuse [14]. Several studies report the importance of facilitating care providers in their handling of elder abuse cases, e.g. creating clear protocols on how to report and manage cases [9, 14, 19, 22]. In this study we consider barriers and facilitators for managing cases of elder abuse as part of the same continuum, e.g. lack of clear protocols for managing cases would be considered a barrier while the existence of such a protocol would be considered a facilitator.

When providing education about elder abuse, it has been found to be important to pay attention to localized needs and provide contact information for relevant local services for victims [12]. Also, the use of interactive teaching techniques has been recommended [12, 23, 24]. Using patient cases and hands-on active learning with real or standardized patients has been well received [12, 24]. One type of interactive training is forum theatre, a form of interactive theatre developed by Augusto Boal [25]. Forum theatre, and a version of it called forum play, has previously been used in health care settings, both with staffs and students, e.g. to counteract abuse in health care and to practice communication skills [26–29].

In conclusion, many health care providers report lacking education about elder abuse [9, 15] and only a few studies have investigated educational interventions about elder abuse directed at health care providers [30]. A recent review about educational interventions for elder abuse in primary care suggests that education needs to focus solely on elder abuse and needs to be comprehensive and concise to realistically allow health care providers to attend sessions. Also, using multiple teaching methods is recommended, including interactive elements, e.g. small-group discussions and role-play to practice communication skills [30]. In this pilot study we test the effectiveness of a comprehensive one-day course about elder abuse, combining theory, group discussions and forum theatre. The latter has been suggested to be an innovative educational method, stimulating reflection and learning within health care [26]. Most previous educational interventions concerning elder abuse in health care have used outcome measures that have not been validated, which is a threat to validity [30]. In this study we used a mixed method approach, combining qualitative interviews and quantitative data collected with a validated questionnaire [10].

### Aim

The aim of this pilot study was to perform initial testing of an educational model aiming at improving health care providers’ preparedness to care for older adults subjected to abuse. We used a mixed method approach to investigate:

1. How health care providers perceived the education and how it influenced a) their propensity to ask older patients questions about abuse and b) their perceived ability to manage the response.
2. Health care providers’ personal and organizational a) sense of responsibility to identify victims, b) barriers and facilitators towards asking questions about elder abuse and managing the response, and how those were affected by the education

## Methods

### Design

This study describes a non-randomized controlled cohort pilot study of an educational model concerning elder abuse, targeting health care providers. A mixed method convergent parallel design was used, i.e. both quantitative and qualitative data were used and they were collected at the same time and given the same importance in analysis [31]. This method was applied to collect different but complementary data to understand the effects of the educational model more comprehensively than when using either method alone. Using complementary data sources is especially beneficial for evaluation studies with limited sample sizes [32], such as ours. In the convergent parallel design, quantitative and qualitative data are collected and analysed separately. Thereafter an interpretation is conducted regarding how the two data sets converge, diverge and relate to each other [31]. In accordance with this we will present the quantitative and qualitative data separately in the methods and results sections, while interpreting and relating the data together in the discussion.

### Participants and setting

In the Swedish health care system, medical interns are physicians who have completed the medical education but are not yet licensed medical practitioners. They complete a structured program working in internal medicine, surgery, psychiatry, emergency medicine and primary care for 18 months. The current study was carried out in a region in southeast Sweden which holds two equivalent programmes for training medical interns. Participants for the intervention group were recruited from one of the programmes while the other program provided participants for the control group. The education took place in October 2020 and was organized as part of the interns’ ordinary educational programme. Initially, all interns employed on one of the two programmes were intended to be offered participation in the education. However, because of restrictions concerning social gatherings during the Covid-19 pandemic, the number of participants had to be limited. An invitation was e-mailed to all the medical interns and the first 43 to respond (out of 58 employees in total) were given the possibility to participate. All interns employed on the other programme were invited to participate in the control group (*n* = 67).

Participants for the qualitative interviews were recruited from the intervention group. We attempted to use purposeful sampling by using the participants’ follow-up evaluations to reach participants with various opinions of the educational day. The possibility for this was, however, limited due to the small number of participants, resulting in a combination of purposeful and convenient sampling.

### Educational model

To facilitate transferal of the acquired competence into practice, the educational model tested was grounded in participants’ own experiences, focused on active participation and contained a mix of different pedagogical methods, i.e. theory, group discussions and forum theatre.

### Theory

The first part of the education (approximately 1 h 10 min) consisted of a lecture given by two of the authors (JS, ML). The lecture started by defining elder abuse and elaborating on its prevalence and health consequences. Two short films illustrating elder abuse cases were interspersed in the lecture to increase awareness of what elder abuse is and elicit emotions. One of the films illustrated a case of psychological abuse and abuse related to physical dependence by an intimate partner while the other film showed a case of neglect by the victim’s son. Thereafter followed some suggestions on how to talk about elder abuse with older patients, a brief introduction to trauma informed care and a presentation on the societal resources available for victims. Written material, including pamphlets about elder abuse directed at staff and patients, was distributed. Also, a screening form that can be used to identify older adults exposed to abuse (REAGERA-S) was introduced [33]. Finally, a brief introduction to motivational interviewing [34] and its applicability in the context of elder abuse was given.

### Case-based group discussions

The second part of the education (approximately 1 h 15 min) consisted of case-based group discussions. Two sets of two short films illustrating patient-health care provider encounters were used as an introduction to the discussions. The first film illustrated a rather unsatisfactory encounter in which an older woman told her health care provider about psychological abuse she endured from her husband. The provider reacted by simply telling the woman what to do (telling the woman to leave her husband), but the provider did not try to understand the complexity of the woman’s situation nor her personal preferences. This was followed by group discussion about challenges in the encounter and how it could be improved. Thereafter a second short film illustrating the same encounter was shown, but this time the health care provider asked open questions and let the older woman talk about the challenges of her situation. Together the provider and older woman started finding strategies to handle her difficulties. Afterwards, group discussions focused on how different strategies taken by the health care providers in the two films changed the outcome of the patient encounter. The same procedure, i.e. two short films with different endings combined with group discussions, was thereafter repeated once again. The topic of the other set of short films was how motivational interviewing techniques can be used to help decision making about life changes and whether to seek help for abusive experiences.

### Forum theatre

The third part of the education (approximately 2 h 30 min) consisted of forum theatre, led by three drama teachers. In forum theatre the spectators are not only observers, but rather spect-actors, urged to participate in the scene [25, 26]. The forum theatre started with the drama teachers acting out a scene showing a problematic health care encounter. The first scene portrayed an older woman about to be discharged from the emergency room after being treated for a broken arm. Plans had been made to initiate home care but when her son, who lived with her, heard about that he became aggressive towards both the health care provider and towards his mother. The health care provider started asking the older woman questions about abuse (with the son present) but she denied such experiences and before she left together with her son, she said that home care was not needed after all. Consequently, the mother’s need for home care was neglected, and questions about abuse were inappropriately posed in the presence of the aggressive son. In the next step of the forum theatre, the same scene was played out again, but now the participants were invited to pause the scene at any time and suggest alternative ways for the health care provider to act. They were also encouraged to take the role of the health care provider in the play themselves, thereby testing the consequences of alternative ways of acting. Discussions between participants about the situation and ways to manage it were also initiated. The scene was repeated several times, and in this way, participants together explored how their actions and reactions could improve the health care encounter, e.g. what happens when the provider talks to the older woman and her son separately? How can the provider gain the older woman’s trust and find ways of helping her? In addition to the aforementioned scene, another one, focusing on how to ask questions about abuse, had been prepared in advanced. Also, participants contributed scenes based on their own experiences, which the drama teachers acted out as improvisations. Due to time restrictions all scenes could not be played out, but they stimulated reflection and discussions among participants about all forms of elder abuse, including difficulties concerning how to identify cases when there are no obvious physical signs of abuse.

### Measurement

The Responding to Elder Abuse in GERiAtric care – Provider questionnaire (The REAGERA-P) was used to evaluate the education quantitatively [10]. It contains questions about personal experiences of talking to patients about abuse, self-efficacy for asking questions about abuse and managing the response, cause for concern as well as known barriers and facilitators for asking questions about abuse. Self-efficacy pertains to a person’s perceived ability to conduct a certain task and theoretically a person with high self-efficacy is more likely to perform that particular task successfully [35]. The development and validation of REAGERA-P is described in detail elsewhere [10]. In brief it has been tested for face validity and comprehensibility through cognitive interviews, while construct and convergent validity was tested in a sample of 154 health care providers. Cronbach’s alpha for the self-efficacy scales was satisfactory (asking questions = 0.75 and managing the response = 0.87) [10]. In this study some new items about sense of responsibility as well as personal and organizational barriers were added to the REAGERA-P (items c and f below). This was done to expand the items that could potentially mediate the effect of the educational model. Comprehensibility of those items was assured by conducting cognitive interviews with five health care providers. All the items in REAGERA-P used for this study are described below and can be found in their entirety as additional file 1. Responses were given on ordinal scales that are presented together with the results in Table 2.

### Items in REAGERA-P concerning study aim 1

- Propensity to ask questions: How many times have you asked older patients questions about abuse in the past six months?
- Perceived ability to manage the response: A five item self-efficacy scale in which respondents were asked to rate their perceived ability to manage different tasks in their work (e.g. helping an older patient subjected to abuse to reach the right body in health care or the right support function in society) on a scale from 0 (= able to manage the task very poorly) to 10 (= able to manage the task very well).

### Items in REAGERA-P concerning study aim 2

iii) Sense of responsibility: To what extent do you feel that a) healthcare services and b) you, in your professional role, have a responsibility to identify older patients who are or have previously been subjected to abuse?
iv) Cause for concern (personal barriers): How concerned are you about the following things when it comes to asking older patients questions about abuse? a) That the patient reacts negatively if I ask questions; b) That the patient-care provider relationship will be negatively impacted if I ask questions; c) That I will not be able to offer the patient a good follow up.
v) Self-efficacy for asking questions (personal facilitator): A three item self-efficacy scale in which respondents were asked to rate their perceived ability to perform different tasks in their work (e.g. asking questions about abuse to an older patient who has no clear indication of now being, or having previously been, subjected to abuse) on a scale from 0 (= able to manage the task very poorly) to 10 (= able to manage the task very well).
vi) Personal and organizational barriers: To what extent do you think that, at your workplace, the following factors prevent you from asking older patients questions about abuse? a) Lack of time; b) My own insufficient awareness of the problem; c) Inadequate routines at the workplace for asking questions; d) Inadequate routines at the workplace for handling the answer.

### Quantitative data collection and analysis

Data was collected at baseline and at 6-month follow-up. Participants in the intervention group filled out a web-based version of the REAGERA-P as the first part of the educational day (October 2020), while the survey was e-mailed to participants in the control group a couple of weeks later. The follow-up survey was e-mailed to both the intervention and control group in April 2021, i.e. 6 months post-intervention. Three reminders were sent at baseline and four at follow-up.

The Pearson’s chi square test, or when appropriate Fisher’s exact test, was used to test for differences in background characteristics of the intervention and control group, as well as for differences between the group of respondents lost to follow-up and retained in the intervention and control group respectively.

Due to the low number of participants, it was not possible to use multivariate statistics or ANOVA to make a comparison between changes in intervention and control group while controlling for other variables. Instead, we tested for univariate differences at baseline and 6-month follow-up.

### Aim 1, Propensity to ask questions

Responses were given on an ordinal scale (none, 1 time, 2–4 times, 5 or more) and two analyses were performed. First, answers were kept on the ordinal level and Wilcoxon’s signed rank test for paired samples was used to compare within-group frequency of asking questions about abuse at baseline and follow-up. Second, data was dichotomized and the McNemar test for paired data was used to compare the proportion of respondents who reported having asked older patients about abuse in the previous 6 months with those who had not or did not remember doing so.

### Aim 1, Perceived ability to manage the response

A sum-score for the five items that constitute self-efficacy for managing the response was created for each participant and a mean score was calculated for the intervention and control group respectively. A paired t-test was used to investigate within group changes on the self-efficacy scale between baseline and follow-up and an independent sample t-test were used to compare difference between the intervention and control group.

### Aim 2, Sense of responsibility, personal and organizational barriers and facilitators

A sum-score for the three items that constitute self-efficacy for asking questions was calculated and a paired t-test was used to compare within group changes in mean between baseline and follow-up while an independent t-test was used to compare differences between the intervention and control group. For ordinal scales, Wilcoxon’s signed rank test for paired samples was used to analyse changes between baseline and follow-up.

### Qualitative data collection and analysis

Semi-structured face-to-face interviews with open-ended questions were conducted by the second author (AM) within five months following the education. The interviewer had not been involved with developing or implementing the educational model. He was, at the time, part of the research team but also a medical intern himself, and was a participatory observer during the education, i.e. he took part in group discussions and forum theatre but was not included in the quantitative data collection. An interview guide with open-ended questions was used (additional file 2). The interview focused on participants’ experiences regarding the education, as well as the kind of support they felt would help them to ask older patients questions about abuse in their clinical practice. All the interviews took place in a secluded room on a university campus, lasted 40–60 min and were recorded and transcribed verbatim.

Data was analysed using qualitative content analysis as described by Graneheim and Lundman [36]. First, two of the authors (AM, KS) read through the transcripts repeatedly to get a sense of the whole. Thereafter, AM extracted the text into meaning units related to the study’s aim. The meaning units were then labelled with codes and sorted into subcategories. An inductive approach was applied, meaning the codes were compared based on similarities and differences before being further sorted into subcategories [36, 37]. The subcategories were then organized into a smaller number of categories. The categories and subcategories were discussed and revised by the same two authors (AM, KS) continuously throughout the process. Thereafter, the results were also discussed with the first author (JS), resulting in agreement on the final four categories. The continuous discussion within the research group aimed at strengthening the validity of the study, not by reaching identical statements, but rather by increasing reflexivity when the authors contested each other’s thoughts and interpretation of results [38].

## Results

### Quantitative results

In total, 43 medical interns signed up for the educational day but only 39 attended. Also, three participants came late or had technical problems answering the questionnaire and were therefore excluded from the study, i.e. 36 interns were eligible for inclusion. One person declined participation, leaving a sample of 35 who answered the baseline questionnaire (response rate 97%). Nineteen participants (54%) in the intervention group were lost to follow-up, leaving a sample of 16 (46%) who participated at both measurement points. Of the 67 medical interns asked to participate in the control group, 20 answered the baseline survey (response rate = 30%). Six participants in the control group (30%) were lost to follow-up, leaving a sample of 14 (70%) who participated at both measurement points. There was no significant difference in background characteristics between the intervention and control group in the sample retained at follow-up. However, the attrition analysis revealed that in the intervention group a higher proportion of those retained at follow-up reported education about violence in a close relationship at baseline, compared to those lost to follow-up (p = 0.03) (Table 1).

**Table 1:** Background characteristics of participants (intervention group n = 16, control group n = 14) and attrition analysis, significant differences at the p < 0.05 level are marked as bold

	**Sample**				**Attrition analysis**							
	**Intervention**		**Control**		**Intervention**				**Control**			
	n = 16		n = 14		Lost to follow-up n = 19		Retained n = 16		Lost to follow up n = 6		Retained n = 14	
	n	%	n	%	N	%	n	%	n	%	n	%
**Sex**												
Female	10	62.5	10	71.4	14	73.7	10	62.5	3	50.0	10	71.4
Male	6	37.5	4	28.6	5	26.3	6	37.5	3	50.0	4	28.6
**Age**												
≤ 34 years	12	75.0	14	100	15	78.9	12	75.0	6	100	14	100
35–49 years	4	25.0	-	-	4	21.1	4	25.0	-	-	-	-
**Medical school training about abuse at baseline**												
No, Do not remember	4	25.0	1	7.1	2	10.5	4	25.0	2	33.3	1	7.1
Yes, violence in close relationships	12	75.0	13	92.9	17	89.5	12	75.0	4	66.7	13	92.9
Yes, elder abuse	2	12.5	4	28.6	4	21.1	2	12.5	1	16.7	4	28.6
**Other training about abuse at baseline**												
No, Do not remember	8	50.0	5	35.7	15	78.9	8	50.0	2	33.3	5	35.7
Yes violence in close relationships	8	50.0	9	64.3	**3**	**15.8**	**8**	**50.0**	4	66.7	9	64.3
Yes, elder abuse	2	12.5	3	21.4	1	5.3	2	12.5	2	33.3	3	21.4
**Asked questions about abuse at baseline**												
No, Do not remember	11	68.8	5	35.7	15	78.9	11	68.8	3	50.0	5	35.7
Yes	5	31.3	9	64.3	4	21.1	5	31.3	3	50.0	9	64.3
**Asked questions about abuse at follow up**												
No, Do not remember	7	43.8	8	57.1								
Yes	9	56.3	6	42.9								

Pearson’s chi square test, or when appropriate Fisher’s exact test, were used to compare differences in background characteristics between intervention and control group at baseline as well as differences between those lost to follow up and those retained in the intervention and control group respectively

### Aim 1

#### Propensity to ask questions

We found a significant increase in the frequency of asking questions in the intervention group at follow-up (p = 0.047), i.e. more respondents reported having asked questions about abuse on several occasions at follow-up. The same pattern was not found for the control group (p = 0.38) (Table 2). There was no significant change concerning the proportion of participants who reported asking patients questions about abuse, but the trend was towards an increase in the intervention group (baseline n = 5; 31%; follow-up n = 9; 56% p = 0.13) and towards a decrease in the control group (baseline n = 9, 64%; follow-up n = 6, 43%, p = 0.25) (Table 1).

**Table 2:** Frequency of asking questions about elder abuse, sense of responsibility and barriers for asking questions

	**Intervention group (n = 16)**						**Control group (n = 14)**					
	**Base-line**	**6 m**	**Rank (n)**			**p**	**Base-line**	**6 m**	**Rank**			**p**
Rank (n)			**Neg**	**Pos**	**Ties**				**Neg**	**Pos**	**Ties**	
**Asked questions about abuse previous 6 months**			1	6	9	**0.047**			4	3	7	0.38
No, Do not remember	11	7					5	8				
One time	1	1					5	3				
2–4 times	3	6					3	1				
5 times or more	1	2					1	2				
**Sense of responsibility**												
** Own responsibility**			1	5	10	0.10						
None	-						-	-	3	3	8	1
Small extent	1	-					1	1				
Some extent	5	3					6	6				
Large extent	10	13					7	7				
**Health care responsibility**			0	4	12	**0.046**			0	2	12	0.16
None	-	-					-	-				
Small extent	1	-					-	-				
Some extent	5	3					8	6				
Large extent	10	13					6	8				
**Perceived Individual level barriers**												
** Own lack of awareness**			2	9	5	**0.04**			1	4	8	0.16
Large extent	7	2					6	3				
Some extent	8	10					6	8				
Small extent	-	4					2	2				
Not at all	1	-					-	-				
**Concern for follow up**			6	3	7	0.19			5	2	7	0.16
Very worried	3	2					2	3				
Rather worried	5	9					4	6				
A little worried	5	5					4	2				
Not at all	3	-					4	3				
**Concern negative reaction**			2	4	10	0.41			3	4	7	0.71
Very worried	-	-					-	-				
Rather worried	3	1					1	2				
A little worried	6	8					8	5				
Not at all	7	7					5	7				
**Concern relationship**			3	5	8	0.37			4	1	9	0.16
Very worried	1	-					-	-				
Rather worried	2	-					1	2				
A little worried	6	10					4	6				
Not at all	7	6					9	6				
**Perceived organizational level barriers**												
** Lack of time**			5	5	6	1.0			3	5	6	0.48
Large extent	6	3					4	4				
Some extent	6	12					8	6				
Small extent	4	1					1	3				
Not at all	-	-					1	1				
**Lack of routines asking**			2	4	10	0.32			1	7	5	**0.03**
Large extent	5	3					4	1				
Some extent	8	9					5	7				
Small extent	3	4					3	5				
Not at all	-	-					1	1				
**Lack of routines managing**			3	5	8	0.37			2	8	4	0.05
Large extent	6	5					7	4				
Some extent	8	7					5	5				
Small extent	2	4					1	3				
Not at all	-	-					1	2				

Changes between baseline and follow up regarding frequency of asking questions about elder abuse as well as changes in sense of responsibility and perceived barriers for asking questions. A positive rank signifies a positive change, i.e., higher sense of responsibility and lower lever of perceived barrier. Significant changes (p < 0.05) are written in bold and have been calculated using Wilcoxon’s signed rank test for paired samples

#### Perceived ability to manage the response

We found a significant increase in self-efficacy between baseline and follow-up for managing the response (p = 0.04) in the intervention group, but not the control group (p = 0.14). The mean difference between baseline and follow up self-efficacy score was 3.8 for the intervention group and 2.8 for the control group, this one-point difference between the groups was not statistically different (p = 0.7) (Table 3).

**Table 3:** Self-efficacy for asking older patients questions about abuse and managing the response

	**Intervention group** **(n = 16)**			**Control group** **(n = 14)**			**Between group comparisons**		
	Mean	SD	P-value	Mean	SD	p-value	Mean	SD	p-value
**Self-efficacy asking questions**									
Baseline	16.3	3.5		19.6	5.3		3.3	1.6	0.05
6 months follow up	19.2	3.0		19.4	5.5		0.2	1.6	0.92
Difference in mean	**2.9**	**5.0**	**0.04**	-0.3	5.2	0.84	3.2	1.9	0.10
**Self-efficacy managing the response**									
Baseline	24.3	7.3		24,9	8.2		0.7	2.8	0.81
6 months follow up	28.1	7.6		27.8	11.4		-0.2	3.5	0.95
Difference in mean	**3.8**	**6.7**	**0.04**	2.8	6.6	0.14	1.0	2.5	0.70

Significant changes (*p* < 0.05) between baseline and 6 months follow-up are written in bold and have been calculated using paired t-tests

### Aim 2

#### Sense of responsibility and internal barriers and facilitators for asking questions about abuse and managing the response

Self-efficacy for asking questions was significantly increased between baseline and follow-up in the intervention group (p = 0.04), but not in the control group (p = 0.84). The mean self-efficacy score increased by 2.9 in the intervention group and decreased by 0.3 in the control group between baseline and follow up. This in-between group difference in mean of 3.2 points was however not significant (p = 0.10) (Table 3). We found no significant changes in either the intervention or control group concerning estimation of own responsibility for asking questions at follow up. However, in both the intervention and control group most respondents reported a high sense of responsibility already at baseline (Table 2). Respondents in the intervention group attributed higher responsibility to the health care organization to ask questions about abuse at follow-up compared to baseline (p = 0.046), which was not seen in the control group (p = 0.16). Respondents in the intervention group were less likely to report their own lack of awareness as a barrier at follow-up (p = 0.04), while there was no such difference in the control group (p = 0.16) (Table 2). There were no significant changes between baseline and follow-up in either the intervention or control group concerning any of the causes for concern when asking questions about abuse. The higher levels of concern (rather worried and very worried) were commonly reported for concern about not being able to provide a proper follow-up, while concerns about negative reactions or negative effects on the patient-provider relationship were commonly reported at the lower levels of concern (not at all or little worried) at both measurement points in both groups (Table 2).

#### Organizational barriers to asking questions about abuse and managing the response

A majority of respondents in both the intervention and control group reported a lack of routines for managing cases as a barrier to some or a large extent at both baseline and follow-up. However, fewer respondents in the control group reported that a lack of routines for asking questions (p = 0.03) and a lack of routines for managing the response (borderline significant, p = 0.052) were barriers to asking questions at follow-up. No significant difference was seen in the intervention group for the same two variables (p = 0.32 and p = 0.37 respectively). No changes were seen for time restraints as a barrier in either the intervention or control group (Table 2).

#### Qualitative results

Four participants were interviewed, all of whom were female. Analysis of the interviews resulted in four categories: Internal processes and new perspectives; Motivational processes; Area of responsibility; Feelings of insecurity and challenges in responding to elder abuse.

#### Internal processes and new perspectives

The participants all described emotional reactions to the content of the educational day, e.g. frustration, discomfort and sadness, but also commitment and curiosity. Experiencing scenarios that were perceived as realistic was an important factor in evoking the emotional responses. Also, an increased awareness of elder abuse was articulated in all interviews. Participants reported finding the subject more important because of the educational day, as well as having more general knowledge and a more comprehensive understanding of the issue. Some participants stated that realizing how common and under-diagnosed elder abuse is, as a result of listening to the theoretical lecture, had made a particular impact on them.

“…I mean, I think the statistics were tough. Yes, that was really the toughest part. […] statistics and this information about… […] how unusual it is for health care providers to ask [about elder abuse], and how many people go unnoticed”. (Participant 4).

Participants expressed that they had made associations between what they were seeing and hearing during the educational day, and their own previous experiences. The portrayal of various abuse-related scenarios, particularly in the videos and in the forum theatre, made the participants reflect on their own experiences of similar situations. This prompted emotional reactions for some, e.g. when realizing they hadn’t asked questions about abuse in situations where they now thought it would have been relevant. In addition, such realizations made them start to apply their acquired knowledge of elder abuse to real life experiences.

“When we saw the videos and other [participants] started to talk about what they’ve seen in wards and primary care offices, you realized ‘Oh my god, so many things have been witnessed’. […] You started to think about what you’ve seen and experienced yourself. That it [elder abuse] is much more common than you think and something you need to open your eyes to…” (Participant 2).

The participants also described that during the day, especially in the forum theatre and group exercises, discussions would continuously arise between participants, which allowed them to share their own thoughts and gain perspectives other than their own. Seeing and hearing their colleagues and the actors play out the scenes in the forum theatre made the participants reflect on their own choice of strategy, as well as providing them with the opportunity to learn lessons from others.

“And the thing about seeing…You learn from how your colleagues are managing it, what words they are using. And when they got stuck, someone else made a contribution. ‘Maybe you could do it [handle the situation] like this?’ Or ‘I would have done it like that’. Then you also learn from your colleagues’ mistakes” (Participant 2).

#### Motivational processes

Most participants expressed that the educational day made them feel involved with and committed to care for victims of elder abuse. For instance, the forum theatre evoked a feeling of involvement and some participants described how they felt compelled to take action to make a difference for the better in the scene that was being played out. An increased awareness of the issue, as well as understanding that their actions could make a difference for the better, was mentioned as important in conveying motivation and interest.

“… having time to talk about the subject and seeing situations… that I hadn’t for example paid attention to before. And feeling that I could be of importance [when encountering victims of elder abuse]. That I could make a difference through my questions or behaviour, that it could improve things for these people… I felt like that created motivation” (Participant 3).

When asked about how their way of working had changed after the educational day, the participants particularly mentioned acting from the position of being more aware of elder abuse, e.g. paying attention to warning signs and symptoms, asking direct questions and not being afraid to ask about abuse, more routinely screening for elder abuse and considering types of abuse other than physical.

“…But I have to say that in those situations when it’s more of an unpleasant atmosphere, I have never had the courage to make a comment on that. But perhaps now…” (Participant 4)

Participants also mentioned being equipped with more tools to manage situations involving elder abuse, such as having the pamphlets that were handed out during the day that contained phone numbers and other contact information to supportive organizations and authorities. They also cited being provided with ideas and inspiration concerning how and when to ask questions about abuse and that they had learned strategies to manage situations where abuse could be suspected. However, some participants emphasized the importance of practising how to ask questions out loud about elder abuse, and that they perceived that there had been too little time to do that during the educational day.

#### Area of responsibility

All the participants expressed the importance of the expectations on them as physicians and on their organizations. Some clearly felt that it was their responsibility to investigate suspected ongoing elder abuse, and to help patients who were suffering from abuse. However, the informants also stated that the extent of their professional responsibility needs to be more clearly defined and explained to them, that they needed to know where their responsibilities toward the patient ended, and other health care professionals’ and social welfare authorities’ responsibilities started. Staying within their area of responsibility was important for the informants and they felt that stepping outside it would be difficult and could put them in a difficult position. For instance, some felt that they might be questioned for prioritizing the management of abuse-related problems if it took time away from their medically related tasks. All the participants also expressed how the patient had to take responsibility as well, e.g. taking action to make a change and accepting help that was offered to them.

“And I believe that if you talk about it more and establish a norm that it’s important that we assist and aid in a certain way, then I think we employees will… work for that and take more responsibility. The way things are now, I don’t feel like it is that way […] It is not our responsibility […] we are supposed to manage other things. And if it is about being exposed [to abuse] in a relationship, it’s the victim themself or other professionals who must take responsibility.” (Participant 3).

#### Feelings of insecurity and challenges in responding to elder abuse:

Some participants felt that the education did not provide them with a solution to an important part of their insecurities, i.e. how to generally manage cases of elder abuse. Some respondents emphasized that they remained unsure of how to act in practice also after the education. Not being able to present the patient with a definite measure or solution left the participants with a feeling of inadequacy. For instance, one participant shared that her insecurities about how to manage the situation could influence her to avoid the subject all together and refrain from asking questions about abuse.

“Often it’s like…What we hear is: ‘Don’t order those lab tests, because it will result in incidental findings.’ And it is a bit the same situation here: ‘Don’t ask that question, because you will have a problem you can’t manage’. I think that is often the case. And I feel like that’s a shame, but sometimes I feel just like that: ‘What do I do with the answer?’.” (Participant 2).

Participants required information about structured and uncomplicated ways to manage situations and sought the possibility to offer concrete measures to the patient, e.g. offering an appointment to someone in charge of follow-ups or making referrals to responsible departments or resources. This would assure the health care provider involved that the issue wouldn’t end with just their contribution, which could provide a sense of security for them. Another approach to lessen their insecurities was to seek support. During the interviews, all the participants expressed a need for support to manage patients subjected to abuse. This support could be e.g. practical routines, consulting more experienced colleagues, social workers or for some a wish to hand over responsibility for following up and managing the situation to someone else.

“…I often feel insufficient not knowing what resources there are […] …Who can this person [the patient] make contact with? Who do we usually send referrals to? […] I wonder if there is something more I could do, that I’m not aware of.” (Participant 3).

#### Integration of quantitative and qualitative results – an overall picture of the educational effects

**Fig. 1:**
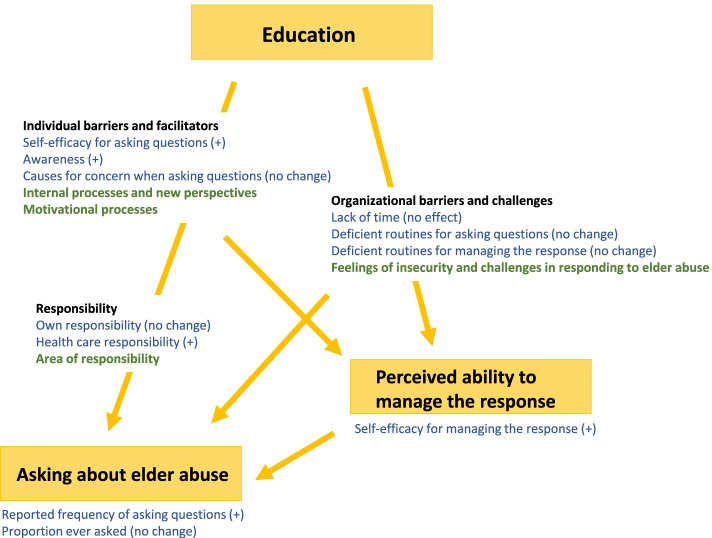
Overview of results. Quantitative data collected at baseline and 6-month follow-up in both the intervention and control groups (blue), and qualitative data collected post-intervention (green). Both the propensity to ask older patients questions about abuse and providers’ perceived ability to manage the response (yellow arrows) were improved in the intervention group at follow-up. The change was possibly mediated by an increased self-efficacy for asking questions and a higher awareness about the issue. Also, the results indicated that the perceived ability to manage the response affected the propensity for asking questions

An overview of both quantitative and qualitative results and how the findings relate to each other can be found in Fig. 1. The results indicate that the model led to an increased propensity to ask older patients questions about abuse and though uncertainties about the best possible response to elder abuse remained, participants also reported higher self-efficacy for managing cases of elder abuse post intervention. The results were possibly mediated by an increased self-efficacy for asking questions and a higher awareness about the issue post intervention, both of which were reflected in the quantitative as well as qualitative results. Also, the results indicated that the perceived ability to manage the response, including availability of guidelines affected the propensity for asking questions. As illustrated in Fig. 1 the quantitative (blue) and qualitative (green) results were in agreement and complement each other, which is further elaborated on in the discussion.

## Discussion

The main aim of this mixed method pilot study was to evaluate an educational model to increase health care providers’ preparedness to care for older adults subjected to abuse. Our findings indicated a positive effect, motivating a full-scale test of the intervention.

### Propensity to ask older patients questions about abuse

Participants in the intervention group reported a significant increase in frequency of asking questions after the intervention, which was not found in the control group. Likewise, in the qualitative interviews, participants expressed that they had changed their practice after the education by, for example, being more attentive to signs of abuse and not hesitating to ask questions. Altogether, this indicates that the education led to an increased propensity to ask older patients questions about abuse. The change was likely in part mediated by a higher commitment to care for victims and an increased awareness of the issue, both of which were clearly articulated in the interviews. Also, the quantitative analysis showed that fewer respondents regarded their own lack of awareness as a barrier to asking questions about abuse at follow-up. Higher awareness after education about elder abuse is unsurprising and has also previously been reported [30, 39]. Some mediators of the increased awareness were articulated in the interviews, e.g. providing knowledge about different aspects of elder abuse during the lectures and case descriptions in the short films and forum theatres.

Another possible mediator for the increased propensity to ask questions about elder abuse was the improved self-efficacy found in the intervention group at follow-up. High self-efficacy indicates feeling more secure about how to perform a certain task, in this case asking questions about abuse and managing the response. Increased self-efficacy for handling intimate partner violence has previously been associated with a higher likelihood of screening for intimate partner violence in health care [40]. One explanation for the higher self-efficacy for asking questions as well as managing the response found at follow-up is likely using forum theatre as a method. The scenes played out functioned as a form of skills training in which participants and drama teachers together explored different ways of acting. Informants articulated in the interviews that the forum theatre and group discussions stimulated reflections on their own way of acting and provided them with new insights and perspectives. Similarly, using interactive teaching techniques in education about elder abuse has previously been found to be more effective than non-interactive teaching interventions [30] and forum theatre has been found useful when practising communication skills in health care education [29].

The mean self-efficacy for asking questions was higher in the control group compared to the intervention group at baseline (borderline significant p = 0.05, Table 3). This finding may have several reasons, e.g., 64% of participants in the control group compared to 31% in the intervention group had experiences of asking older patients questions about abuse at baseline and a larger proportion of participants in the control group compared to intervention group reported previous education about both violence in close relationships and elder abuse (Table 1). These differences between the groups were not statistically significant – potentially because of the low number of participants—but might partially explain the difference in self-efficacy between the two groups at baseline. The differences also indicate that potential confounding factors may be unevenly distributed between the two groups and therefor the within group differences pre- and post-intervention may be a more reliable measure of the effect of the education than the in-between groups analysis. Our within group analysis revealed that the self-efficacy increased significantly at follow up in the intervention group, but not in the control group, which indicates an effect of the education. However, this needs to be verified in a full-scale test of the model in which multivariate analyses including potential confounding factors can be included and more reliable in-between group comparisons can be made.

### Perceived ability to manage the response

We found a significant increase in self-efficacy for managing the response in the intervention group at follow-up, which was not found in the control group. However, in the qualitative interviews, the informants expressed that they remained insecure about how to manage cases of elder abuse, and that this instilled in them a feeling of inadequacy. One potential explanation for this discrepancy between the quantitative and qualitative results is that the education might have opened the eyes of some participants to the deficiency in clear guidelines and organizational preparedness to care for victims of elder abuse. In the interviews, the informants expressed a need for support, as well as structured and concrete measures to offer the patient, as a means to lessen their insecurities and to provide the best possible care for victims. To date, however, there are no evidence-based programmes that can be generally applied to victims of elder abuse [41, 42]. This is in part due to a paucity of studies on the subject, but also a reflection on the complexity of the issue, where all victims have individual needs [43, 44]. Hence, the sought-after clear-cut way of managing the response is perhaps not possible to achieve. However, though still feeling insecure, informants reported in the interviews that they felt better equipped with tools and strategies to detect and manage elder abuse after the education than before and as previously mentioned, self-efficacy for managing the response increased at follow up. Altogether, this indicates that the education might provide health care providers with skills to administer the best possible care within the available societal support system.

One theme recurring in the qualitative interviews was the sense of responsibility. The participants expressed in the interviews that they perceived health care organizations and themselves as health care providers as responsible for detecting and managing elder abuse. This is concurrent with the quantitative results, in which most respondents reported a high sense of responsibility both at baseline and follow-up. There were no significant changes concerning estimation of own responsibility for identifying victims at baseline compared to follow-up. This could possibly be attributed to a ceiling effect. Already at baseline 10 out of 16 participants rated both health care system responsibility and own responsibility as the highest level (large extent). In a future study, response categories need to be changed to avoid a ceiling effect. This is important, considering that a strong sense of professional responsibility has previously been associated with having experiences of talking to older patients about abuse [9].

Though feeling responsible, the participants elaborated in the interviews on uncertainties about their role in managing cases of elder abuse, e.g. what they were expected to do and what they should leave for others to do. The Swedish National Board of Health and Welfare states in its directive that whenever a patient shows signs or symptoms that indicate abuse, health care providers have a responsibility to ask questions and provide contact with relevant societal resources [45]. Even so, participants expressed concerns in the interview that it might not be appropriate to prioritize managing abuse-related issues, because it was not considered part of the operational tasks of their clinic. This seems to be an important barrier to getting involved in cases of elder abuse and hence needs to be more clearly addressed in a future full-scale test of the education. Only medical interns, i.e. early-career physicians, were included in this study, which might have contributed to uncertainties about what the professional role entails. However, similar to our findings, previous research has suggested that health care providers do rely on other occupations, e.g. social workers, to manage elder abuse issues [14].

### Limited ability to manage the response as a barrier to asking questions about abuse

The perceived ability to manage cases of elder abuse was also found to be related to the propensity to ask questions, e.g. one of the participants interviewed mentioned that insecurities about how to manage cases might discourage her from enquiring about elder abuse altogether. Likewise, concerns about not being able to provide a proper follow-up and lack of routines for managing cases were reported as barriers to asking questions about abuse by many participants in both the intervention and control group. These barriers were unfortunately not affected by the intervention.

In the control group we found an unexpected significant decrease in considering lack of routines for asking questions and lack of routines for managing the response as barriers to asking questions. At approximately the same time as the intervention was carried out, new guidelines on how to manage violence in close relationships, were introduced in the region where the study was carried out. The section about elder abuse was considerably extended in the new version of the guidelines and more practical advice on how to manage cases were introduced. There is no obvious explanation for why this should have affected the control group more than the intervention group, but it is possible that—for some unknown reason—more participants in the control group compared to the intervention group were aware of the new guidelines and hence were less inclined to consider lack of routines as a barrier for asking questions and managing the response.

### Implications for future education about elder abuse

The benefits of using different pedagogical strategies to evoke interest and commitment towards caring for older adults subjected to abuse was a recurrent subject in the interviews, e.g. one participant stated that the statistics were the most striking part, while another underlined that the films with patient cases elicited the most emotions and interest. Building on previous experiences and learning from each other in group discussions, and especially forum theatre, were also repeatedly mentioned as important elements of the education. In a future full-scale test of the education, it might be beneficial to integrate theory and group discussions even more, to further stimulate collaborative learning and exchange of ideas.

More emphasis during the education needs to be directed at what to do when an older adult reports experiences of abuse. As mentioned before, there is no single solution for how cases of elder abuse should be handled. There are, however, some general principles that could be further outlined during the intervention, e.g. what trauma-informed care response entails for this patient group [46]. Also, more emphasis should be directed at explaining the societal resources that are available for victims. To further make visible what can be done in individual cases, a brief remark after each forum theatre could also be made about potential ways of helping in each specific case. In that way participants are provided with examples of how to manage a few role-model cases, which they can then return to and contemplate when faced with cases in clinical practice.

### Limitations

In this pilot study, the number of participants was low, and a rather large proportion were not retained at follow-up. Also, only female participants agreed to participate in the qualitative interviews. Hence, results should be interpreted with caution and all results need to be replicated in a full-scale study. However, the quantitative and qualitative results point in the same direction, towards a positive effect, which increases the validity of the results. Those who were retained in the intervention group were more likely than those lost to follow-up to report previous education concerning violence in close relationships at baseline, possibly indicating a higher interest in the issue. It is possible that the intervention was less effective among participants lost to follow-up considering they might have been less committed to the issue to begin with. Also, the group of participants was homogeneous in that they were young, early-career physicians. It is probable that the results would have been different if the group had been more heterogeneous regarding e.g. profession or clinical experiences. This will be explored in a future full-scale test of the model that is on-going and will provide the opportunity to test for differences e.g. between different professional categories [47].

The qualitative interviews were in some cases conducted several months after the intervention took place, which means there is a risk of recall bias. However, the qualitative data was rich in content, which indicates that limitation of recall was not of great importance for the results. The author who conducted the qualitative interviews (AM) was a participatory observer during the education. This can be labelled a form of insider research and might have affected the results. It is possible that the interviewer’s own experience and thoughts about the education affected the questions asked during the interviews, as well as interpretation of the data. These risks were handled by using an interview guide to steer the interviews and by letting another researcher (KS) with no relation to the participants take part in the analysis. Also, continuous discussions of coding and tentative subcategories and categories within the research group stimulated reflexivity in the analysis, reducing the risk of reproducing preconceived ideas. It has previously been suggested that when insider and outsider researchers collaborate, this provides a possibility to gain a more profound understanding of the phenomenon studied [48, 49].

## Conclusion

In this mixed method pilot study, we tested an educational model aiming at improving health care providers’ preparedness to care for older adults subjected to abuse. Results indicate that the education led to an increased propensity to ask questions about abuse, possibly through raising awareness and commitment to identifying and helping in cases of elder abuse, as well as by increasing participants’ self-efficacy for asking questions. The results concerning managing the response to elder abuse were more ambiguous: self-efficacy for managing cases of elder abuse increased at follow-up, but both the quantitative and qualitative results indicated that uncertainties about how to manage cases of elder abuse remained. Hence, one important lesson learned was that in future tests of the model, more focus needs to be put on how to manage cases of elder abuse. A full-scale test of the model is currently being conducted. A study protocol has been published [47] and the study is registered at clinicaltrials.gov (register no NCT05065281).

## Supplementary Information

**Additional  file 1.** REAGERA-P.

**Additional  file 2.** Interview guide.

## Data Availability

The datasets generated and analysed during the current study are not publicly available and are not available in its entity from the corresponding author on request. This is due to the small sample size, which would make it impossible to maintain participant privacy and confidentiality if made publicly available. Data not including background characteristics or other information that could potentially be used to identify participants is available from the corresponding author upon reasonable request.

## Abbreviation

REAGERA-P: Responding to Elder Abuse in GERiAtric care – Provider questionnaire
