# Supplementary material for: Testing an educational intervention to improve health care providers’ preparedness to care for victims of elder abuse: a mixed method pilot study
Source: BMC Med Educ. 2022 Aug 3;22:597. doi: 10.1186/s12909-022-03653-8 (PMC9351204; doi:10.1186/s12909-022-03653-8)
Supplement: Supplementary file 1 — Additional file 1. REAGERA-P. [file 12909_2022_3653_MOESM1_ESM.pdf]

REAGERA-P

(Responding to Elder Abuse in GERiAtric care – Provider questionnaire)

A. Background characteristics

1. **Are you**
   - Female
   - Male
   - Other

2. **How old are you?**
   - Up to 34 years old
   - 35-49 years old
   - 50 years old or older

3. **What is your current profession?**
   - Assistant nurse
   - Nurse
   - Physician
   - Other

4. **How long have you worked in your current profession?**
   - Less than one year
   - One to five years
   - Five or more years

5. **How long have you worked at your current workplace?**
   - Less than one year
   - One to five years
   - Five or more years

6. **What is your primary workplace?**
   - Internal medicine department, ward or clinic
   - Geriatric department, ward or clinic
   - A health care centre
   - I rotate between different workplaces since I am a medical intern (house officer)

7. **In your education, did you receive training on violence in close relationships (regardless of age) or elder abuse?** (Multiple answers possible)
   - Yes, elder abuse
   - Yes, violence in close relationships
   - No
   - Do not remember

8. **Did you at any other time receive training on violence in close relationships (regardless of age) or elder abuse?** (Multiple answers possible)
   - Yes, elder abuse
   - Yes, violence in close relationships
   - No
   - Do not remember

9. **To what extent do you feel that it is OK at your workplace to question the managers how you work, or to point out shortcomings in the activities?**
   - to a large extent
   - To a somewhat large extent
   - To some extent
   - To a small extent

10. **To what extent do you feel that the employees at your workplace seek help from each other if there is something they do not know how to do, or that they have the courage to say if they feel uncertain about something or have made a mistake?**
    - to a large extent
    - To a somewhat large extent
    - To some extent
    - To a small extent

Abuse of an older person includes:

- **physical, emotional and sexual violence or abuse**
- **financially exploitation**
- **neglect**

By older, we mean individuals over 65 years of age.

By "asking questions about abuse", we mean that you directly ask if the patient has been treated badly or subjected to some kind of abuse. Accordingly, we do not mean general questions about circumstances at home or how they are doing.

This applies to the entire questionnaire.

B. Case vignette

You will now be asked to read a patient case. In your work, what would you think about asking this patient questions about abuse in different phases of your contact?

Gunnel, aged 77, is admitted to the hospital due to a deterioration of her COPD. Her breathing rapidly improves, but Gunnel instead complains a lot about abdominal pain. She has sought care for this several times both at the health care centre and the emergency ward, but the pain does not improve. She previously underwent a thorough investigation, including gastroscopy, without any explanation for the symptoms being found.

11. How likely is it, based solely on this information, that you ask Gunnel questions about abuse?

- Not at all likely
- Not particularly likely
- Somewhat likely
- Very likely

In the conversation, it comes forth that Gunnel in recent years has sought care on multiple occasions with different symptoms, but no good explanation has been found for her symptoms. Among other things, she was treated for chest pain that was not deemed to be cardiac related, and she has had very troublesome back pain for an unclear reason.

12. Based on the information you now have access to, how likely is it that you ask Gunnel questions about abuse?

- Not at all likely
- Not particularly likely
- Somewhat likely
- Very likely

Before the next time you see Gunnel, you see in the medical records that she has been depressed periodically. Last year, she received in-patient care over 24 hours because she had taken too many of her antidepressive pills. In the medical record, it says that the overdose was probably happened by mistake, but that the circumstances were a little unclear. After that care episode, Gunnel received Apodos so that it would not happen again.

13. Based on the information you now have access to, how likely is it that you ask Gunnel questions about abuse?

- Not at all likely
- Not particularly likely
- Somewhat likely
- Very likely

Gunnel says that she is single and lives in a villa. She has handled it well so far, but she says that she would need home-help services now to be able to manage everything. Gunnel has a son who lives in the same city and he has financial problems and therefore lives with Gunnel now and then. When you see Gunnel, you ask if she likes having her son living with her sometimes. Gunnel answers vaguely and evasively. A few days later, a needs assessment is done and the son then says that he thinks it is unnecessary to spend money on the home-help services, and Gunnel agrees. Afterwards, you meet Gunnel alone again.

14. **Based on the information you now have access to, how likely is it that you ask Gunnel questions about abuse?**
    - Not at all likely
    - Not particularly likely
    - Somewhat likely
    - Very likely

You now also examine Gunnel again and note something that you had not seen before. She has older bruises on both upper arms. When you ask what happened, Gunnel tries to joke the question away and says that she does not know, but that she might have “happened to bump into something”.

15. **Based on the information you now have access to, how likely is it that you ask Gunnel questions about abuse?**
    - Not at all likely
    - Not particularly likely
    - Somewhat likely
    - Very likely

C. Cause for concern

How concerned are you about the following things when it comes to asking older patients questions about abuse?

16. **That the patient reacts negatively if I ask questions**
    - Not at all concerned
    - A little concerned
    - Somewhat concerned
    - Very concerned

17. **That the patient-care provider relationship will be negatively impacted if I ask questions**
    - Not at all concerned
    - A little concerned
    - Somewhat concerned
    - Very concerned

18. **That I will not be able to offer the patient a good follow-up**
    - Not at all concerned
    - A little concerned
    - Somewhat concerned
    - Very concerned

D. Self-efficacy

19. At present, how would you manage to do the following things in your work?

a. Asking questions about abuse to an older patient who has clear indications of now being, or having previously been, subjected to abuse
b. Asking questions about abuse to an older patient who has no clear indications of now being, or having previously been, subjected to abuse
c. Ensuring you are able to ask questions about abuse in private to an older patient who has a relative who insists on being present during all contact
d. In conversation, providing support to an older patient who tells about abuse
e. Helping an older patient subjected to abuse to reach the right body in healthcare, or to the right support function in society
f. Helping an older patient subjected to abuse to make a report to the police or social services
g. Helping and supporting an older patient subjected to abuse, who does not currently want to change his or her situation
h. Handling the meeting with an older patient who says no to questions about abuse, but where you still have strong suspicions that the patient is subjected to abuse

Responses are given on a 11 point scale ranging from:
0 = Would manage it very poorly
10 = Would manage it very well

E. Own previous experiences

20. To what extent do you feel that you can assess the likelihood that an older patient was subjected to abuse without having to ask specific questions?

- To a large extent
- To a somewhat large extent
- To some extent
- To a small extent

21. How many times in the past six months has an older patient spontaneously told you about experiencing abuse, without you asking questions about it?

- None
- Once
- 2-4 times
- 5 times or more
- Do not remember

22. How many times have you asked older patients questions about abuse in the past six months?

- None
- Once
- 2-4 times
- 5 times or more
- Do not remember

23. How many times did the questions lead to an older patient telling about abuse that he or she experienced?

- None
- Once
- 2-4 times
- 5 times or more

24. Have you at any time had lingering suspicions that the patient is or has been subjected to abuse even though he or she has denied it when you asked questions about it?

- No
- Yes, once
- Yes, several times

25. Feel free to tell a little about one such situation:

26. Think about the last time an older patient told you about abuse. *To what extent do you think that the patient received a good follow-up?*

- To a large extent
- To a somewhat large extent
- To some extent
- To a small extent
- The patient was deemed not to need follow-up
- The patient was offered follow-up, but turned it down
- Cannot assess how the follow-up turned out

27. Feel free to tell more about the handling here:

28. In the past six months, have you had suspicions that an older patient was subjected to abuse, but did not ask questions about it?

- I have not had any such suspicions
- I have had suspicions, but did not ask any questions
- I have always asked questions if I had suspicions
- Do not remember

29. What was it that led to you not asking questions? (Multiple answers possible)

- The suspicions were not strong enough
- I was uncertain about how to ask questions
- I was uncertain about how to handle the answer
- I have too little professional experience to ask questions
- I thought it was somebody else's responsibility to ask questions
- I raised the issue with colleagues and somebody else asked questions
- I raised the issue with colleagues, but it did not lead to anyone asking questions
- Another reason, namely:

F. Sense of responsibility

30. To what extent do you feel that healthcare services have a responsibility to identify older patients who are or have been subjected to abuse?

- To a large extent
- To some extent
- To a small extent
- Not at all

31. To what extent do you feel that you, in your professional role, have a responsibility to identify older patients who are or have been subjected to abuse?

- To a large extent
- To some extent
- To a small extent
- Not at all

32. What professional category at your workplace do you feel has the greatest responsibility to identify older patients who are or have been subjected to abuse?

- Physician
- Nurse
- Assistant nurse
- Psychologist or counsellor
- Other professional categories
- Everyone has an equal responsibility

G. Attitude towards routinely asking questions

To what extent do you feel that you at your workplace should strive to routinely ask questions about abuse to the following patient groups?

33. All older patients with certain diagnoses or symptoms (e.g. depression or indistinct pain)

- Not at all
- To a small extent
- To some extent
- To a large extent

34. All older patients who seek care with symptoms for which no medical explanation is found

- Not at all
- To a small extent
- To some extent
- To a large extent

35. All older patients

- Not at all
- To a small extent
- To some extent
- To a large extent

H. Perceived barriers

To what extent do you think that, at your workplace, the following factors prevent you from asking older patients questions about abuse?

36. Lack of time
- Not at all
- To a small extent
- To some extent
- To a large extent

37. My own insufficient awareness of the problem
- Not at all
- To a small extent
- To some extent
- To a large extent

38. Inadequate routines at the workplace for asking questions
- Not at all
- To a small extent
- To some extent
- To a large extent

39. Inadequate routines at the workplace for handling the answer
- Not at all
- To a small extent
- To some extent
- To a large extent

I. Own exposure to violence

Below are some concluding questions of a more personal nature. They are about your own possible experiences of having been subjected to abuse in life. We are asking the questions to be able to investigate if there is an association between what one has personally experienced in life and how one relates to older patients who have been subjected to abuse. As for other questions in the questionnaire, your responses are personal, but all analyses are done on a group level and that is also how the results will be presented.

It is common to have been subjected to some kind of abuse during life. If this is your case and you have a need for support and help to process this, please turn to one of the support services that are described in the folder you received in connection with the training day.

If you do not want to answer these questions, you may opt to pass by them one by one.

40. Have you yourself, as a child or as an adult, been subjected to any kind of physical abuse?
Such as being beaten, kicked, forcibly held or subjected to other physical violence that you perceived as frightening

- No
- Yes, as a child (<18 years)
- Yes, as an adult (≥18 years)

40b. Who subjected you to abuse as an adult (≥18 years)?

- A partner or former partner
- A family member or relative
- Another person I knew
- A completely unknown person

40c. Who subjected you to abuse as a child (<18 years)?

- A partner or former partner
- A family member or relative
- Another person I knew
- A completely unknown person

41. Have you yourself, as a child or as an adult, been subjected to any kind of sexual abuse?
Such as somebody touching your body against your will or forcing you to perform sexual acts

- No
- Yes, as a child (<18 years)
- Yes, as an adult (≥18 years)

41b. Who subjected you to abuse as an adult (≥18 years)?

- A partner or former partner
- A family member or relative
- Another person I knew
- A completely unknown person

41c. Who subjected you to abuse as a child (<18 years)?

- A partner or former partner
- A family member or relative
- Another person I knew
- A completely unknown person

42. Have you yourself, as a child or as an adult, been subjected to any kind of emotional abuse? For example, that somebody repeatedly degraded you, humiliated you or tried to limit your contact with others or decide what you may and may not do

- No
- Yes, as a child (<18 years)
- Yes, as an adult (≥18 years)

42b. Who subjected you to abuse as an adult (≥18 years)?

- A partner or former partner
- A family member or relative
- Another person I knew
- A completely unknown person

42c. Who subjected you to abuse as a child (<18 years)?

- A partner or former partner
- A family member or relative
- Another person I knew
- A completely unknown person

43. Have you yourself, as a child or at an adult age, been subjected to any kind of financial or material abuse? For example, that somebody exploited you financially or took control of your finances

- No
- Yes, as a child (<18 years)
- Yes, as an adult (≥18 years)

43b. Who subjected you to abuse as an adult (≥18 years)?

- A partner or former partner
- A family member or relative
- Another person I knew
- A completely unknown person

43c. Who subjected you to abuse as a child (<18 years)?

- A partner or former partner
- A family member or relative
- Another person I knew
- A completely unknown person

This questionnaire is a further development of the Responding to Elder Abuse in GERiAtric care – provider questionnaire, previously published under a creative common attribution 4.0 license.

Reference: Simmons, J., Wenemark, M. & Ludvigsson, M. Development and validation of REAGERA-P, a new questionnaire to evaluate health care provider preparedness to identify and manage elder abuse. BMC Health Serv Res 21, 473 (2021). https://doi.org/10.1186/s12913-021-06469-2.

Compared to the original publication items Nos 6, 9, 10, 20, 24-43 were added. Items Nos 18-25 from the original publication was not included.
