# Supplementary material for: Testing an educational intervention to improve health care providers’ preparedness to care for victims of elder abuse: a mixed method pilot study
Source: BMC Med Educ. 2022 Aug 3;22:597. doi: 10.1186/s12909-022-03653-8 (PMC9351204; doi:10.1186/s12909-022-03653-8)
Supplement: Supplementary file 2 — Additional file 2. Interview guide. [file 12909_2022_3653_MOESM2_ESM.pdf]

Interview guide

- Tell me your experiences of the educational day
- Do you think the content of the education met your needs? In what ways? What was missing?
- In what ways were you able to practice your skills concerning meeting older adults subjected to abuse during the education? What things felt important to practice?
- What was your experience of the different parts of the education?
    - Theory
    - Short films and group discussions
    - Forum theatre
        - How did you feel before the forum theatre took place? How did you experience participating in the forum theatre?
        - How did you perceive the patient cases used in the forum theatre?
        - How would you describe what happened in the room during the forum theatre? Within in the group? Inside you?
        - Did the forum theatre contribute to the education beyond what the theory and group discussions contributed?

- What did you experience as positive during the day?
- What did you experience as less positive?
- How can the education be improved?
- What parts of the education evoked the most feeling in you? In what way? Why?
- What is the most important thing you bring with you from the education?
- How will you work when meeting older patient in the future? How is that different from your way of working before the education?

- Tell me about your experiences of meeting older patients subjected to abuse
- Tell me about your experiences of situations in which you had suspicions that an older patient had been (or were at risk of being) subjected to abuse? What was it that evoked your suspicions? What did you do then?
    - If no experiences: Do you think that you during your work have met older patients subjected to abuse? What would you do if you got such suspicions?

- In what kind of situations would you ask your patients questions about abuse?
- How do you perceive your own responsibility to identify elder abuse cases? How do you perceive other health care professions' responsibility to identify elder abuse cases?
- How can physicians become better at identifying elder abuse?

- How do you perceive your ability to care for older adults subjected to abuse?
    - Pertaining to asking questions? How could that ability improve?
    - Pertaining to managing the response? How could that ability improve?
